# Gonadotropins in Keratoconus: The Unexpected Suspects

**DOI:** 10.3390/cells8121494

**Published:** 2019-11-22

**Authors:** Dimitrios Karamichos, Brayden Barrientez, Sarah Nicholas, Symon Ma, Lyly Van, Sashia Bak-Nielsen, Jesper Hjortdal

**Affiliations:** 1 Department of Cell Biology, University of Oklahoma Health Sciences Center, Oklahoma City, 73104 OK, USA; 2 Department of Ophthalmology/Dean McGee Eye Institute, University of Oklahoma Health Sciences Center, Oklahoma City, 73104 OK, USA; Brayden-Barrientez@ouhsc.edu (B.B.); Sarah-Nicholas@ouhsc.edu (S.N.); symonntma@gmail.com (S.M.); Lyly-Van@ouhsc.edu (L.V.); 3 Department of Ophthalmology, Aarhus University Hospital, Palle Juul-Jensens Boulevard 167, 8200 Aarhus N, Denmark; sashia.bak@gmail.com (S.B.-N.); jesper.hjortdal@dadlnet.dk (J.H.)

**Keywords:** keratoconus, gonadotropins, hormonal imbalance, corneal dystrophy

## Abstract

Keratoconus (KC) is the most common ectatic corneal disease with a significant visual acuity burden. The actual burden is intangible given that KC can disrupt daily activities (reading, driving, and various career paths). Despite decades of research and clinical studies, the etiology, onset, and pathobiology of KC remain a mystery. The purpose of this study was to investigate the role of gonadotropins in KC. We recruited 86 KC patients (63 males, 23 female), and 45 healthy controls (22 male, 23 female). Plasma samples were collected and analyzed using an enzyme-linked immunosorbent assay. Corneal stromal cells from KC and healthy controls, and human epithelial corneal cells, were also investigated for gonadotropin-related markers. Our results show significant alterations of LH/FSH in KCs, compared to healthy controls. Our data also reveals, for the first time, the existence of gonadotropins and their receptors in KC. Our study is the first to demonstrate the role of LH/FSH in KCs, and expand the list of organs known to express gonadotropins, or their receptors, to include the human cornea. Our findings suggest that the human cornea is capable of responding to gonadotropins, and propose an intriguing mechanism for the onset and/or progression of KC.

## 1. Introduction

Keratoconus (KC) is a complex, multifactorial, corneal disease that remains a mystery, in the context of its onset, rate of progression, and underlying pathobiology [1,2]. Progressive ectasia, corneal thinning, and scarring are the hallmarks of KC, known to affect both males and females [1]. Prevalence has been increasing steadily, going from 1:2000 back in the 1980s to 1:350 in the 2000s worldwide [3,4,5,6], perhaps due to technological advancements and better diagnostics.

Hunting for an etiology for the appearance and progression of KC, studies have been reporting a slew of contributing factors including genetics [7], environmental conditions [1,8], eye rubbing [9], and hormonal imbalances [10]. Recently, our group has been focusing on hormonal imbalances and their role on KC. We have reported significant changes in androgens and estrogens in KC patients, when compared to healthy subjects. We found significant upregulation of dehydroepiandrosterone sulfate (DHEA-S), while estrone and estriol were downregulated in plasma and saliva samples, from KC patients [11]. Surprisingly, these changes were apparent independent of age, gender, or severity of KC. In the same cohort of KCs, we discovered that a hormonally regulated protein, prolactin-induced protein (PIP), was significantly downregulated in all KCs, leading us to believe that PIP is a strong KC biomarker and a measurable indicator of KC [12]. However, the signaling mechanisms about how hormonal imbalances and PIP can tell the KC story are unknown. So, how can hormonal imbalances explain the large geographic variability, the gender dominance inconsistencies, or the unpredictable rate of progression? The current study sought to investigate and delineate the mechanisms underlying hormonal imbalances in KCs.

The idea that sex hormones play a role in KC is not new. Estrogen and androgen receptors are present in the human cornea, though their role is uncertain [13]. The role of sex hormones in causing changes in the cornea during pregnancy has long been documented, as hormonal fluctuations result in corneal alterations including increases in corneal volume, central corneal thickness, and curvature [14]. Due to these hormonal changes, progression in KC has been documented in pregnant women during pregnancy and beyond, extending to six months postpartum [15]. Therefore, understanding the mechanisms of hormonal regulation in KC is fundamental to determining dysfunction, and can directly affect people’s lives.

The pituitary gland is responsible for secretion of various endocrine signaling hormones [16] with the anterior pituitary producing follicle-stimulating hormone (FSH) and luteinizing hormone (LH), which ultimately control the sex hormones (androgens and estrogens) [17]. FSH is a gonadotropin, a glycoprotein polypeptide hormone that is synthesized and secreted by the gonadotropic cells of the anterior pituitary gland [16]. FSH is known to regulate the development, growth, pubertal maturation, and reproductive processes of the human body [18]. LH is also produced by gonadotropic cells in the anterior pituitary gland [17]. In females, LH triggers ovulation and development of the corpus luteum, whereas in males, LH stimulates Leydig cell production of testosterone [19]. Both FSH and LH are vital endocrine hormones, and their involvement in KC has never been investigated. Based on our recent findings and the role of sex hormones in KC, we sought to investigate the levels and modulation of LH/FSH in KC patients, both systemically and within the corneal microenvironment (i.e., cellular level).

Our data shows significant modulation of LH and FSH levels in KCs, when compared to their healthy counterparts, in vivo, suggesting a systemic component that up until now remained unexplored. Furthermore, we surprisingly found expression of LH, as well as the LH/FSH receptors, by human corneal stromal cells, and the FSH receptor by human corneal epithelial cells, in vitro. These novel findings should qualify the human cornea, for the first time ever, as an extragonadal tissue and place it in the same category as previously identified tissues: skin [20], breast [21], uterus [22] and adrenals [23].

Based on our findings, as well as our previous studies, we would like to propose a new concept for the onset of KC. We propose that KC is a systemic disease initiated by hormonal imbalances regulated by the anterior pituitary at very early ages (if not at birth) and manifests around puberty due to the long-term exposure of the cornea to these imbalances. Further investigation and clarification of LH/FSH-mediated signaling events is necessary, together with consideration of cross talk between pathways. Such studies will allow us to understand the roots of KC and begin to develop preventative strategies.

## 2. Materials and Methods

### 2.1. Participants, Ethical Approval, Consent, and Inclusion/Exclusion Criteria

This study adhered to the tenets of the Declaration of Helsinki. The studies were approved by The Central Denmark Region Committees on Health Research Ethics (protocol number: 1-10-72-127-16), and by the Institutional Review Board (IRB)/Ethics committee at the University of Oklahoma Health Sciences Center, Dean McGee Eye Institute (IRB protocol #3450). All participants underwent a thorough ophthalmologic examination including Pentacam HR, refraction, and slit lamp examination to confirm the KC diagnosis and exclude any other ophthalmic diseases or dystrophies. Healthy controls underwent a similar ophthalmologic examination to ensure that they did not have KC or other eye diseases or dystrophies. Patients with any present or previous cancer as well as serious systemic diseases were excluded. However, patients with well-treated hypertension, well-treated hypercholesterolemia and well-treated asthma, were included. To the best of our knowledge, female patients included in the study were not under contraceptive control. All participants signed a written informed consent before participation. All laboratory analyses of human biological fluids were performed blindly, to minimize bias. Table 1 shows KC severity groups, which were defined according to maximum corneal curvature (Kmax; worst/most severe eye), as well as demographic characteristics of KC patients and healthy controls.

### 2.2. Plasma Sample Collection

EDTA-coated 10 mL tubes (BD vacutainer^®^, Franklin Lakes, NJ, USA) were used for blood samples from all participants. EDTA-coated tubes were gently inverted to secure the mixture of whole blood and were centrifuged for 10 min at 1300× *g* at 4 °C to separate plasma [24]. Plasma samples were then stored at −80 °C, following transfer to sterile microfuge tubes, until further analyses.

### 2.3. Plasma ELISA

Hormone levels in plasma samples were detected using the following commercial immunoassay kits: Human Luteinizing Hormone ELISA Kit (Abcam, Cambridge, MA, USA) and Human Follicle Stimulating Hormone ELISA Kit (Abcam, Cambridge, MA, USA). Briefly, 50 µL of prepared standards and samples were loaded in duplicate into the appropriate wells, followed by addition of 100 µL of enzyme conjugate reagent into each well. ELISA plates were incubated, in the dark, on a shaker at room temperature at 200 RPM for 45 min. Following rinsing with deionized water, 100 µL of TMB reagent was added into each well and gently mixed for 10 s. The plate was then incubated in the dark on a shaker at room temperature at 200 RPM for 20 min. Next, 100 µL of stop solution was added to each well and gently mixed for 30 s. Within 15 min of mixing, the samples were measured in a plate reader at 450 nm. A curve-fitting statistical software was used to plot a 4-parameter logistic curve fit to the standards and then calculate results for all of the samples.

### 2.4. Corneal Tissue Processing and Cell Isolation

Healthy corneas were obtained from the National Disease Research Interchange (NDRI). KC corneas were obtained from individuals immediately following corneal transplantation. Inclusion/exclusion criteria for healthy controls required absence of ophthalmic disease, diabetes, or infectious conditions. Tissue from KC patients who had previously undergone collagen crosslinking was excluded.

Stromal Cells: Corneal stromal cells were isolated from healthy (HCFs) and KC (HKCs) corneas. Both HCFs and HKCs were isolated as previously described [25,26]. Briefly, using a surgical scalpel, the corneal epithelium and endothelium were removed. The corneal stroma was then washed in sterile PBS, cut into small pieces (approximately 2 × 2 mm), and placed into flasks; the cells were then allowed to adhere. Explants were grown for 2–4 weeks at 37 °C/5% CO_2_/95% relative humidity, using Eagle’s Minimum Essential Media (EMEM) supplemented with 10% fetal bovine serum (Atlanta Biologicals, Flowery Branch, GA, USA), and antibiotic/antimycotic (Anti/Anti, Life Technologies, Grand Island, NY, USA). Once confluent, cells were isolated following trypsinization, subcultured, or frozen using standard cryoprotective protocols.

Epithelial Cells: Telomerase-immortalized human corneal epithelial cells (HCECs) were kindly provided by Dr. Pablo Argueso (Schepens Eye Research Institute/Mass. Eye and Ear, Boston, MA, USA), and stored in liquid nitrogen until further analysis [27].

### 2.5. Cell Cultures and In Vitro Models

Three-dimensional constructs - Stromal Cells: HCFs and HKCs were plated at a density of 1 × 10^6^ cells/well on six-well size polycarbonate membrane inserts with 0.4-μm pores (VWR, Radnor, PA, USA). The cells were cultured in EMEM containing 10% FBS, 1% antibiotic, and stimulated with a stable Vitamin C derivative (0.5 mM 2-*O*-α-d-glucopyranosyl-l-ascorbic acid: Sigma-Aldrich, St. Louis, MO, USA). Cultures were grown for a total of 4 weeks and fresh media was supplied every other day for the duration of the study. Protein extraction and further analysis occurred at the 4-week timepoint.

Two-dimensional conventional cultures - Epithelial Cells: Cells were brought up from liquid nitrogen and cultured in a T75 flask in epithelial media, consisting of Keratinocyte Growth Media 2 PromoCell (VWR, Radnor, PA, USA), Penicillin-Streptomycin Solution (ThermoFisher, Rockford, IL, USA), and CaCl_2_ (Sigma-Aldrich, St. Louis, MO, USA). Once confluent, cells were removed via trypsinization and seeded in a 6-well plate at 1 × 10^6^ cells/well in epithelial media. Cells were cultured at 37 °C for 24 h before protein was extracted [27,28].

### 2.6. Protein Extraction and Western Blot Analysis

All cultures were collected and washed twice with PBS, and total protein was isolated using 1X radioimmunoprecipitation assay (RIPA) buffer (50 mM Tris pH 8, 150 mM NaCl, 1% Triton X-100, and 0.1% SDS) containing a protease inhibitor cocktail (Sigma-Aldrich, St. Louis, MO, USA). Cell lysates were centrifuged at 4 °C, and the supernatant was isolated and subjected to a Pierce BCA Protein assay (ThermoScientific, Rockford, IL, USA) for protein concentration quantification. Novex 4–20% Tris-glycine mini WedgeWell format 12-well gels (FisherScientific, Hampton, NH, USA) were electrophoresed at 130 V for 1.5 h, and transferred onto a nitrocellulose membrane at 100 V for 1 h on ice. Blots were blocked in 5% dry milk (Great Value, Bentonville, AR, USA) for 1 h, at room temperature, with shaking. After preparing primary antibodies in a 1:1000 dilution, blots were incubated in mouse monoclonal antibody FSH (FisherScientific, Hampton, NH, USA) and the following primary rabbit polyclonal antibodies: β-Actin (Abcam, Cambridge, MA, USA), FSH-R (Abcam, Cambridge, MA, USA), LH-R (Abcam, Cambridge, MA, USA), and LH (Bioss, Woburn, MA, USA). The samples were rocked overnight at 4 °C, and washed with TBST before being incubated in the diluted secondary antibody goat anti-mouse IgG (H+L) AlexaFluor Plus 555 (ThermoFisher Scientific, Rockford, IL, USA) while rocking for 1 h at room temperature. After washing with TBST, the blot was allow to dry and imaged using a ChemiDoc-It^2^ imager. Results were analyzed by normalizing values to the expression of housekeeping antibody β-Actin (Abcam, Cambridge, MA, USA).

### 2.7. Statistical Analysis

Data analysis and presentation were executed using GraphPad Prism 7.0 (GraphPad Software, Inc., La Jolla, CA, USA). Data are presented as mean +/− SEM using bar plots. Dot-box plots and representative western blots are provided as Supplementary Figures. Using one-way ANOVA, and t-test where necessary, a value of *p* < 0.05 was considered significant. The n number for each experiment is listed in the appropriate legend and/or the bar plot.

## 3. Results

### 3.1. LH/FSH in Healthy Controls and KCs

**Figure 1:**
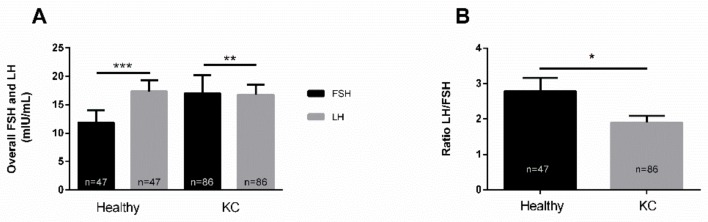
Expression of LH and FSH, and the LH/FSH ratio in healthy (n = 47) and KC (n = 86) blood (plasma) samples. (**A**) Overall levels for both LH and FSH, and (**B**) LH/FSH ratio in healthy controls and KCs. * *p* < 0.05, ** *p* < 0.01, *** *p* < 0.001. (See Supplementary Materials—Figure S1).

The expression of LH and FSH in human plasma samples from KC patients and healthy controls was determined using ELISAs. Human plasma is easily accessible and is commonly used in both clinical and biological studies [12,29]. FSH levels were elevated in KCs, but not significantly, when compared to healthy controls (Figure 1A). Similarly, LH levels, showed no significant differences between healthy and KCs (Figure 1A). In healthy controls, LH levels were higher than those of FSH (Figure 1A), while in KCs, the opposite was observed (Figure 1A).

The circulating levels of LH/FSH ratio is deemed to be a more relevant measure, and has been investigated as a potential marker in polycystic ovary syndrome [30]. Our findings showed a significantly lower LH/FSH ratio in KCs when compared to healthy controls (Figure 1B). This is the first evidence, ever reported, suggesting a correlation between KC pathology and circulating LH/FSH.

### 3.2. Gender Dependence

**Figure 2:**
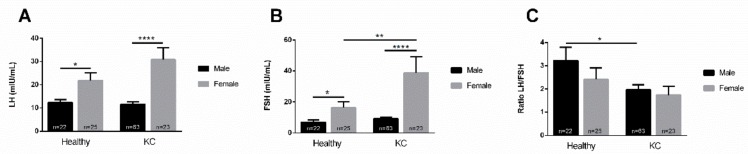
Gender-dependent expression of LH, FSH, and LH/FSH ratio in healthy and KC blood (plasma) samples. (**A**) Healthy male (n = 22), healthy female (n = 25), KC male (n = 63) and KC female (n = 23) LH levels; (**B**) healthy male (n = 22), healthy female (n = 25), KC male (n = 63) and KC female (n = 23) FSH levels; and (**C**) the male and female LH/FSH ratio, in healthy controls and KCs. * *p* < 0.05, ** *p* < 0.01, **** *p* < 0.0001. (See Supplementary Materials—Figure S2).

Depending on the geographical region and the cohort of patients, gender bias in KC is at best inconsistent. We investigated the levels of LH and FSH in the context of male and female patients. Our data shows that both LH (Figure 2A) and FSH (Figure 2B) levels were higher in females, as compared to males. This was true for both healthy controls and KCs, although this disparity was much more prominent in KCs. Furthermore, FSH in female KCs was significantly higher than the FSH levels in healthy females (Figure 2B).

Regarding the LH/FSH ratio, KCs showed a lower ratio in both males and females (Figure 2C), when compared to their healthy counterparts. Specifically, the LH/FSH ratio in KC females tended to be lower than in healthy females, but not significantly (Figure 2C). However, the LH/FSH ratio in KC males was significantly lower compared to healthy males (Figure 2C).

LH and FSH bind to receptors in the testis and ovary, regulating gonadal function by promoting sex steroid production and gametogenesis. There is a known gender-bias for the two hormones, which manifests further downstream of the sex hormones. This would suggest that any changes seen here are more related to KC gender pathology rather an LH and FSH gender bias. Therefore, the LH/FSH ratio could be a measure used to “bypass” all the inconsistencies and discrepancies seen between genders, in KC, in different geographical regions.

### 3.3. Age Dependence

**Figure 3:**
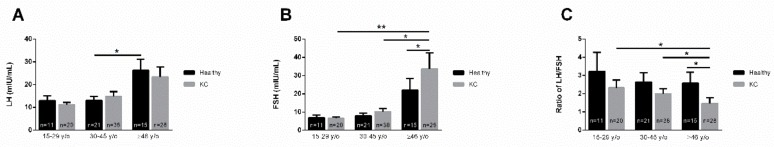
Age-dependent expression of LH and FSH, and the LH/FSH ratio, in healthy and KC blood (plasma) samples. Three age groups were investigated: Healthy 15–29 y/o (n = 11), KC 15–29 y/o (n = 20), Healthy 30–45 y/o (n = 21), KC 30–45 y/o (n = 38), Healthy ≥ 46 y/o (n = 15) and KC ≥ 46 y/o (n = 28). (**A**) LH levels in healthy controls and KCs, per age group, (**B**) FSH levels in healthy controls and KCs, per age group, and (**C**) the LH/FSH ratio in healthy controls and KCs, per age group. * *p* < 0.05, ** *p* < 0.01. (See Supplementary Materials—Figure S3).

KC is known to appear around puberty and arrest itself by the age of 40 or 50. We therefore investigated the plasma levels of both LH and FSH in three age groups: 15–29 y/o, 30–45 y/o, and 46–older. Both LH and FSH remained unchanged, in KCs and healthy controls, for age groups 15–29 y/o and 30–45 y/o (Figure 3A). However, at age group 46 or older, significant upregulation of LH (Figure 3A) or FSH (Figure 3B) was seen in healthy controls and KCs, respectively. The LH/FSH ratio showed significant progressive dowregulation in KCs, from the younger to the older population, whereas the ratio remained unchanged in healthy controls (Figure 3C).

**Figure 4:**
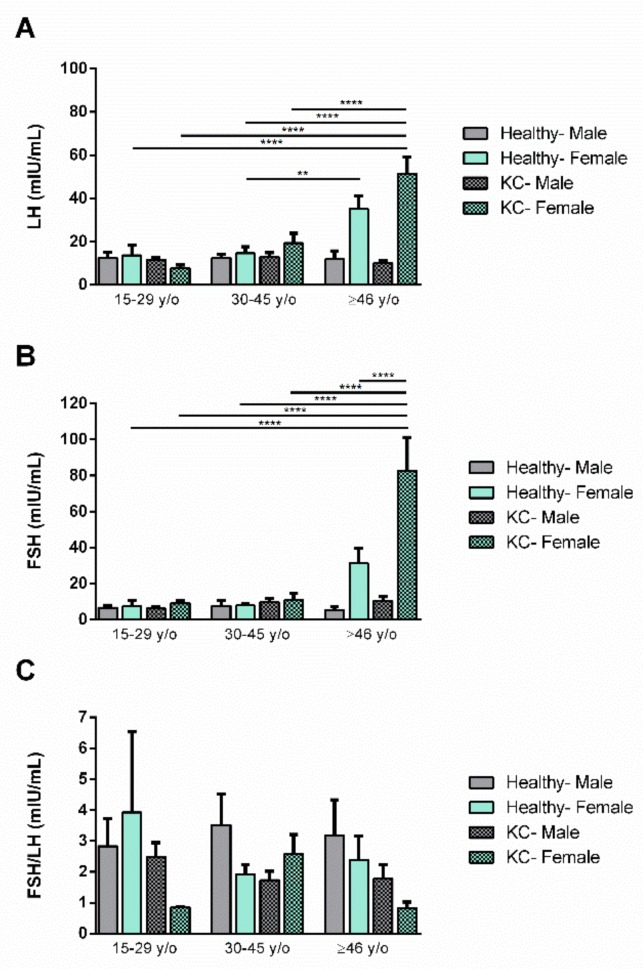
Age effect as a function of gender expression of LH and FSH, and the LH/FSH ratio, in healthy and KC blood (plasma) samples. The following groups were investigated: Healthy Males 15–29 y/o (n = 7), Healthy Females 15–29 y/o (n = 4), KC Males 15–29 y/o (n = 18), KC Females 15–29 y/o (n = 2), Healthy Males 30–45 y/o (n = 10), Healthy Females 30–45 y/o (n = 12), KC Males 30–45 y/o (n = 26), KC Females 30–45 y/o (n = 12), Healthy Males ≥ 46 y/o (n = 5), Healthy Females ≥ 46 y/o (n = 9), KC males ≥ 46 y/o (n = 19), KC Females ≥ 46 y/o (n = 9). (**A**) LH levels in healthy controls and KCs, (**B**) FSH levels in healthy controls and KCs, and (**C**) the LH/FSH ratio in healthy controls and KCs, per age group. ** *p* < 0.01, **** *p* < 0.0001. (See Supplementary Materials—Figure S4).

We further investigated the effects of age as it relates to gender, in KCs and healthy controls. Both male/female LH and FSH were unchanged for age groups 15–29 y/o and 30–45 y/o (Figure 4A,B, respectively), in both KCs and healthy controls. However, age group 46–older showed increased levels of LH and FSH in KC females and healthy females. This suggests that the increase we saw for total LH and FSH (Figure 1) is largely due to the female population.

The LH/FSH ratio analysis showed no significant changes. However, it is interesting to highlight that the lowest LH/FSH ratio was seen in KC females at the 15–29 y/o and ≥46 y/o age groups.

### 3.4. KC Severity

**Figure 5:**
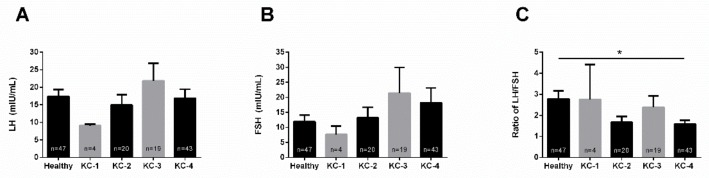
Severity-dependent expression of LH and FSH, and the LH/FSH ratio in healthy (n = 47) and KC (n = 86) blood (plasma) samples. Severity grades were defined based on the Kmax: KC-1 (n = 4), KC-2 (n = 20), KC-3 (n = 19) and KC-4 (n = 43). (**A**) LH levels in healthy controls and KCs, across all severities, (**B**) FSH levels in healthy controls and KCs, across all severities, and (**C**) the LH/FSH ratio in healthy controls and KCs, across all severities. * *p* < 0.05. (See Supplementary Materials—Figure S5).

We investigated LH and FSH levels based on KC severity. Severity groups were defined according to maximum corneal curvature (Kmax). LH (Figure 5A) and FSH (Figure 5B) levels were largely unaffected among the severity grades (KC1 through KC4), as well as when compared to healthy controls. The LH/FSH ratio was significantly downregulated with the KC4 group (Figure 5C), but remained unchanged for the rest of the severity groups. There may be a correlation between LH/FSH and KC severity, however, larger cohorts will need to be investigated in order to safely determine this relationship.

### 3.5. KC Treatment

**Figure 6:**
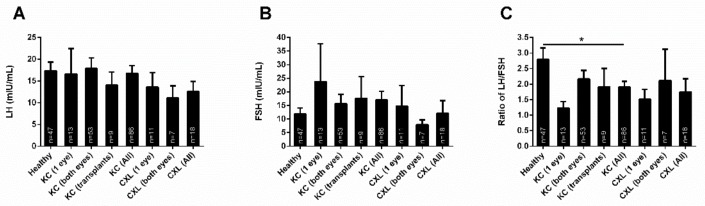
Expression of LH and FSH, and the LH/FSH ratio in healthy (n = 47) and KC (n = 86) blood (plasma) samples. Seven different groups were compared: (1) KC on one eye (n = 13), (2) KC on both eyes (n = 53), (3) KCs with corneal transplants (n = 9), (4) All KCs independent of treatment(s) (n = 86), (5) KCs with collagen crosslinking on one eye (n = 11), and (6) KCs with collagen crosslinking on both eyes (n = 7), and 7) All KCs with collagen crosslinking treatment, independent of the number of eyes (n = 18). (**A**) LH levels, (**B**) FSH levels, and (**C**) the LH/FSH ratio. * *p* < 0.05. (See Supplementary Materials—Figure S6).

In order to determine if KC presence and/or type of treatment(s) affected the levels of either LH or FSH, we investigated the following groups: Individuals with: (1) KC on one eye, (2) KC on both eyes, (3) KCs with corneal transplants, (4) All KCs independent of treatment(s), (5) KCs with collagen crosslinking on one eye, (6) KCs with collagen crosslinking on both eyes, and (7) All KCs with collagen crosslinking treament, independent of the number of eyes. All seven groups were plotted and compared against the Healthy controls (Figure 6). There was no significant differences in the LH (Figure 6A) or FSH (Figure 6B) levels between the groups/treatments tested and their Healthy counterparts. Similarly, there were no significant differences among groups/treatments or compared to Healthy, in the LH/FSH ratio. LH/FSH ratio in KC(All) was significantly downregulated, when compared to Healthy, as shown in Figure 1B.

Correlation between treatments and whether or not LH/FSH levels are altered over time, following KC treatment, would require a longitudinal cohort study.

### 3.6. Gonadotropins and Extragonadal Tissue

**Figure 7:**
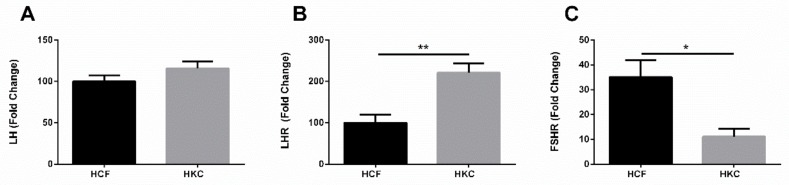
Expression of LH, LHR, and FSHR in HCFs and HKCs. (**A**) LH expression in vitro, (**B**) LHR expression in vitro, and (**C**) FSHR expression in vitro. * *p* < 0.05, ** *p* < 0.01. (See Supplementary Materials—Figure S7).

Based on our findings with the plasma samples, we investigated the expression of LH, FSH, and their receptors (LHR and FSHR), utilizing our established 3D in vitro model. Figure 7A shows expression of LH in both HCFs and HKCs, with no significant differences. FSH was not expressed in either HCFs or HKCs. Expression of LHR was significantly upregulated in HKCs (Figure 7B), whereas FSHR (Figure 7C) was significantly downregulated, when compared to HCFs.

**Figure 8:**
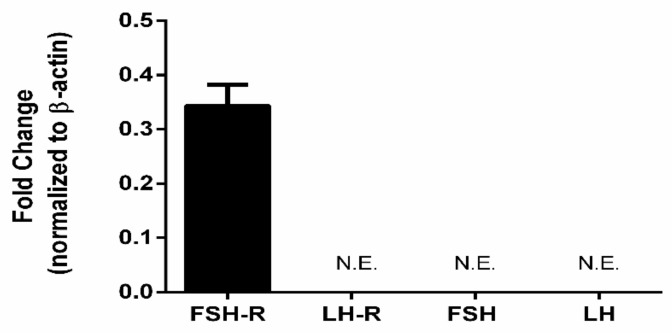
Expression of FSHR, LHR, FSH, and LH in human corneal epithelial cells. FSHR was the only one expressed by the epithelial cells. (See Supplementary Materials—Figure S8).

To our knowledge, this is the first study to demonstrate the presence of LH/LHR/FSHR in the human corneal stroma. These findings raise the possibility that gonadotropins may play a key role in the onset and/or progression of KC. At the very least, corneal stromal cells have the appropriate receptors to respond to gonadotropin-initiated signals. In order to further determine whether the gonadotropin presence is only in the corneal stroma or extends to other cellular layers within the cornea, we tested established epithelial cells in vitro. Figure 8 shows that HCECs only expressed FSH-R. We found no expression for LH, FSH, or LH-R.

## 4. Discussion

The human eye, for a long time, has been considered as “neutral” in terms of gender bias. There has never been enough evidence to suggest that ocular physiology or pathology is affected by sex. However, today we know that sex plays a key role in ocular health and disease, driven by the presence (or absence) of sex hormones.

Sex hormones circulate through the blood stream, and profoundly affect the physiology of multiple organs and tissues [31]. Their effects are highly dependent on the receptors present in the various target tissues/cells. In the human cornea, sex hormones are present in the tear fluid, secreted by the migratory plasma cells and the secretory epithelium of the lacrimal gland [32,33]. Inevitably, with the tears in direct contact with the ocular surface, any hormonal fluctuations may alter corneal homeostasis and therefore visual acuity. Aqueous humor, located posteriorly to the cornea, can also influence corneal homeostasis.

The first reported link between sex hormones and ocular diseases was made by Henrik Sjogren in 1930 when he described a correlation between hormonal changes in women and dry eye syndrome (DES) [34]. In more recent studies, the importance and role of sex hormones in ocular structures has been better characterized. In the context of KC, hormonal regulation has always been suspected due to the correlation with puberty [35] and pregnancy [15], as well as a trend towards stabilization after menopause [36]. However, clinical studies have been lacking. Kahan et al. reported higher levels of thyroxine hormone in tears of KC patients, when compared to healthy counterparts [37]. Thyroxine levels in blood (serum) of these patients, however, showed no significant changes. Thanos and co-authors also found higher levels of tear thyroxine in KCs, as well as high expression of thyroxine receptors in the KC stromal cells, when compared to healthy controls [2]. While very limited literature exists on thyroxine and KC, it is known to play a role in metabolism and physiological function of the cornea as well as other tissues [2]. Furthermore, in a recent case study, published by Lee et al. [38], a 17-year-old girl underwent partial thyroidectomy without thyroid hormone replacement therapy with a normal baseline ophthalmic exam. At the age of 29 years, KC appeared on the right eye, highlighting the potential association of KC and thyroid regulation. Leptin, an energy expenditure hormone, was also investigated in situ by Aydemir et al. [39]. The study found mild staining intensity of the KC corneal cells, which was, however, not significantly different from healthy controls. Leptin is another understudied hormone in KC, although it might be an interesting target of investigation given that leptin levels rise during pregnancy and fall after childbirth [40]. It is possible that these changes in leptin levels could partially explain the association between pregnant women and KC progression; however, there is limited evidence at this time [39]. Our group recently reported significant elevation of DHEA-S and decreased estrone and estriol levels, in KC tears, saliva, and blood (plasma) [12]. Rather surprisingly, our data showed that modulation of these sex hormones were independent of age, gender and KC severity, suggesting that something more fundamental is altered during the disease state. In light of the accumulated data on sex hormones and KC, we sought to delineate and unravel the KC etiology.

KC is a very peculiar disease, in that gender and age prevalence seem to be highly variable depending on the cohort(s) examined. We therefore suspected that androgens/estrogens are malfunctioning, in KCs, due to a dysfunction further upstream in the hormone cascade. Our data shows significant modulation of LH and/or FSH hormones in KCs, when compared to healthy controls. To our knowledge the two hormones, and/or their ratio, have never been investigated, or reported, in the context of KC. Based on our findings, we postulate that FSH and/or LH dysfunction is the cause for the abnormal modulation of DHEA-S, estriol, and estrone, we see further downstream [10].

The obvious question then becomes, how do these hormonal changes seen in KCs, affect the corneal microenvironment, and do the corneal cells have the mechanisms/receptors to receive and respond to these stimuli. The expression of hormone receptors in the KC cornea is rather unexplored. In fact, very little evidence exists. Yin et al. reported a reduction of progesterone receptor (PR) and higher expression of androgen receptor (AR), where Ayan et al. reported higher estrogen receptor (ER) and AR expressions in KC corneas. Our group reported higher mRNA levels of ER and lower AR, in KC-derived corneal stromal cells compared to healthy controls [41]. It is highly possible that these receptors are critical in maintaining the KC corneal homeostasis and are regulated by the abnormal hormone levels found in KC tears (DHEA-S, estrone, and estriol), as previously reported [12]. How sex hormone receptors are regulated in vivo and how the hormone levels in the tear fluid interact with these receptors, is unclear. The affinity, however, could vary between tissues and cell types, and therefore the mechanisms in KCs could be further complicated.

Hinged on our in vivo data, we examined whether the corneal stroma cells express LH/FSH and their corresponding receptors (LHR/FSHR). Traditionally, expression of these gonadotropins is thought to be restricted to gonadal tissue. However, recent studies have shown at least LH is expressed in some peripheral tissues [20,21,22,23]. FSH seems to be more exclusive to the gonadal tissues. Incredibly, both HCFs and HKCs expressed LH, but not FSH, with no significant differences between the two cell types. Furthermore, we found expression of both LH-R and FSH-R, with LHR upregulated and FSHR downregulated, in HKCs when compared to HCFs. Not surprisingly, our findings were different in HCECs. HCECs only expressed FSHR, while no expression was detected for LH, FSH, or LHR.

Clarification of the LH/FSH-mediated intracellular signaling, for all corneal cell types, is critical together with understanding of potential cross-talk and compensatory mechanisms between cells and pathways. At this stage, it is unclear what the role of these receptors is in the human cornea. One possibility would be that expression is mediated by the LH and/or FSH present within the corneal tissue, the tear fluid, and/or the aqueous humor, subsequently leading to either a protective or adverse effect in the KC cornea. Another possibility would be that these receptors play a role in KC progression via a specific downstream signaling cascade, ultimately leading to visual impairment. Whether the human cornea is also capable of producing LH or FSH, which can then bind to the receptors and function as an autocrine or paracrine mechanism, is also unknown. It is important to note that, in females, the menstrual cycle should be monitored in KCs in order to better understand the role of LH/FSH. The menstrual cycle state is something that is currently ignored in the clinics and therefore information is lacking. The fact that changes were observed in the male population, whom do not have fluctuations of LH and FSH, gives us confidence that KC is in fact linked to these hormones. One of the limitations with KC is the lack of early-disease tissue availability. However, unravelling the cellular and molecular mechanisms using cells/tissue from severe KC is more beneficial if we are to move towards the development of novel treatment modalities.

Overall, our in vitro and in vivo studies may provide a new paradigm for KC management and treatment.

## 5. Conclusions

Our proposed mode of action for KC development is via the regulation of gonadotropins, at the pituitary gland, and their receptors within the human corneal microenvironment, leading to changes of ECM proteins, which are critical in maintaining corneal homeostasis. It is plausible that LH/FSH dysfunctions appear at birth, or at very early stages in life, and KC is a manifestation of prolonged hormonal abnormalities derived from the anterior pituitary. Clearly, the proposed mechanism requires further clinical studies in order to be confirmed. Nevertheless, these mechanisms could explain many of the characteristics and discrepancies of the disease.

## Figures and Tables

**Table 1:** Demographic characteristics of the Keratoconus and the healthy control group.

Sub-Groups	Healthy Controls	Keratoconus
**Gender**		
Male	22	63
Female	25	23
**Age Groups**		
1: 15-29y/o	11	20
2: 30-45y/o	21	38
3: >46y/o	15	28
**Severity**		
1: <48D	--	4
2: ≥48-53D	--	20
3: ≥53-58D	--	19
4: ≥58D	--	43
**Treatment**		
KC (One eye)	--	13
KC (Both eyes)	--	53
KC (All)	--	86
CXL (One eye)	--	11
CXL (Both eyes)	--	7
CXL (All)	--	18
Transplant	--	9

## Supplementary Materials

The following are available online at https://www.mdpi.com/2073-4409/8/12/1494/s1, Figure S1: Overall LH/FSH expression, Figure S2: Gender-dependent LH/FSH expression, Figure S3: Age-dependent LH/FSH expression, Figure S4: Age effect as a function of gender expression of LH/FSH, Figure S5: Severity-dependent LH/FSH expression, Figure S6: Treatment-dependent LH/FSH expression, Figure S7: HCF/HKC-Protein expression of LH, LHR, FSHR, and β-actin, Figure S8: HCEC-Protein expression of FSHR and β-actin.
