# Supplementary material for: Gonadotropins in Keratoconus: The Unexpected Suspects
Source: Cells. 2019 Nov 22;8(12):1494. doi: 10.3390/cells8121494 (PMC6953013; doi:10.3390/cells8121494)
Supplement: Supplementary file 1 [file cells-08-01494-s001.pdf]

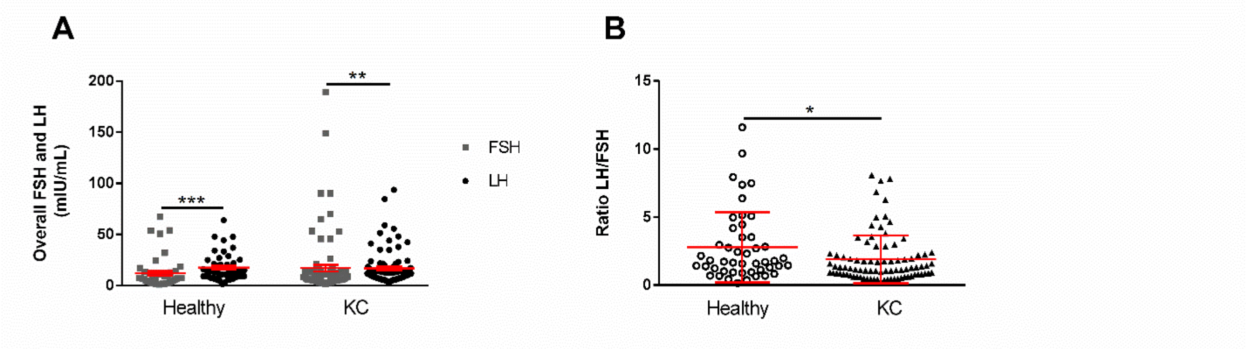

Figure S1. Expression of LH, FSH, and LH/FSH ratio in Healthy (n=47) and KC (n=86) blood (plasma) samples. (**A**) Overall levels for both LH and FSH, and (**B**) LH/FSH ratio in Healthy and KCs. *p<0.05, **p<0.01, ***p<0.001.

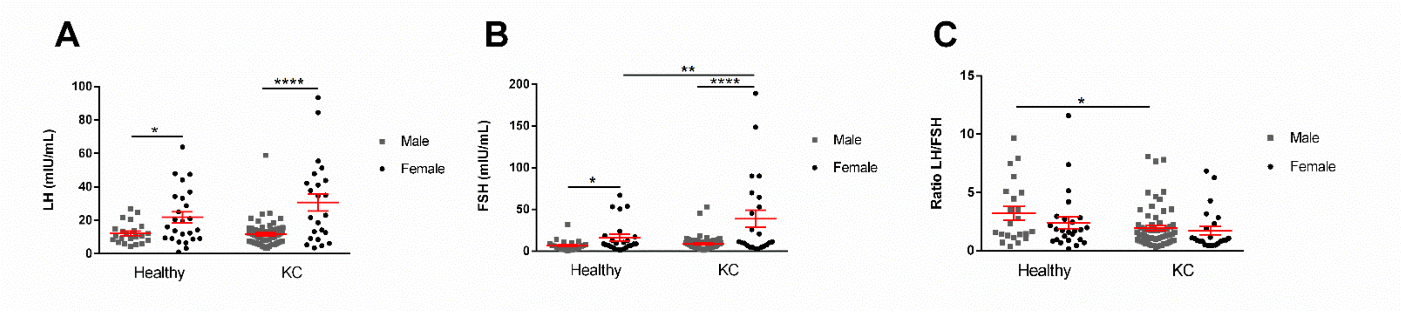

Figure S2. Gender-dependent expression of LH, FSH, and LH/FSH ratio in Healthy and KC blood (plasma) samples. (**A**) Healthy Male (n=22), Healthy Female (n=25), KC Male (n=63) and KC Female (n=23) LH levels, (**B**) Healthy Male (n=22), Healthy Female (n=25), KC Male (n=63) and KC Female (n=23) FSH levels, and (**C**) the Male and Female LH/FSH ratio, in Healthy and KCs. *p< 0.05, **p<0.01, ****p<0.0001.

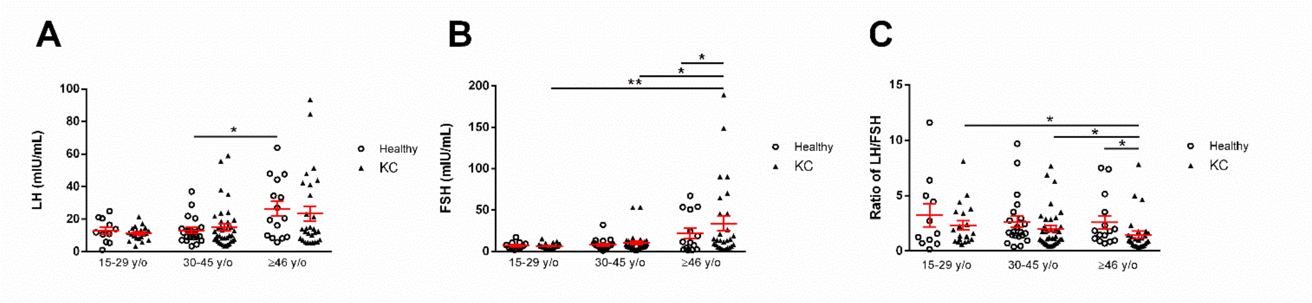

Figure S3. Age-dependent expression of LH, FSH, and LH/FSH ratio in Healthy and KC blood (plasma) samples. Three age groups were investigated: Healthy 15–29 y/o (n=11), KC 15–29 y/o (n=20), Healthy 30–45 y/o (n=21), KC 30–45 y/o (n=38), Healthy ≥ 46 y/o (n=15) and KC ≥ 46 y/o (n=28). (**A**) LH levels in Heathy and KCs, per age group, (**B**) FSH levels in Healthy and KCs, per age group, and (**C**) LH/FSH ratio in Healthy and KCs, per age group. *p<0.05, **p<0.01.

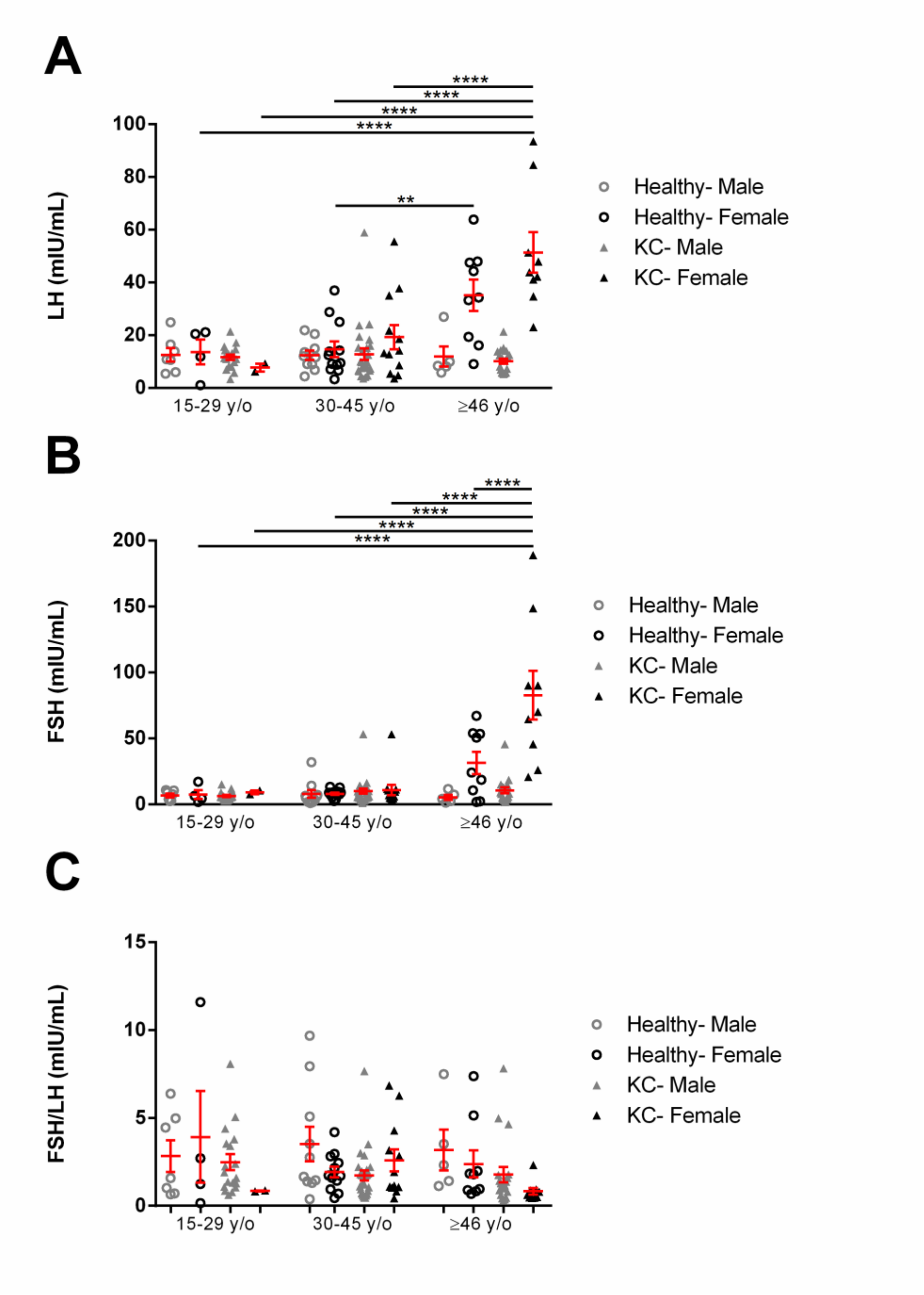

Figure S4. Age effect as a function of gender expression of LH, FSH, and LH/FSH ratio in Healthy and KC blood (plasma) samples. The following groups were investigated: Healthy Males 15–29 y/o (n=7), Healthy Females 15–29 y/o (n=4), KC Males 15–29 y/o (n=18), KC Females 15–29 y/o (n=2), Healthy Males 30–45 y/o (n=10), Healthy Females 30-45y/o (n=12), KC Males 30-45y/o (n=26), KC Females 30–45 y/o (n=12), Healthy Males ≥46 y/o (n=5), Healthy Females ≥46 y/o (n=9), KC males ≥46 y/o (n=19), KC Females ≥46 y/o (n=9). (**A**) LH levels in Heathy and KCs, (**B**) FSH levels in Healthy and KCs and (**C**) LH/FSH ratio in Healthy and KCs, per age group. ****$p<0.0001$.

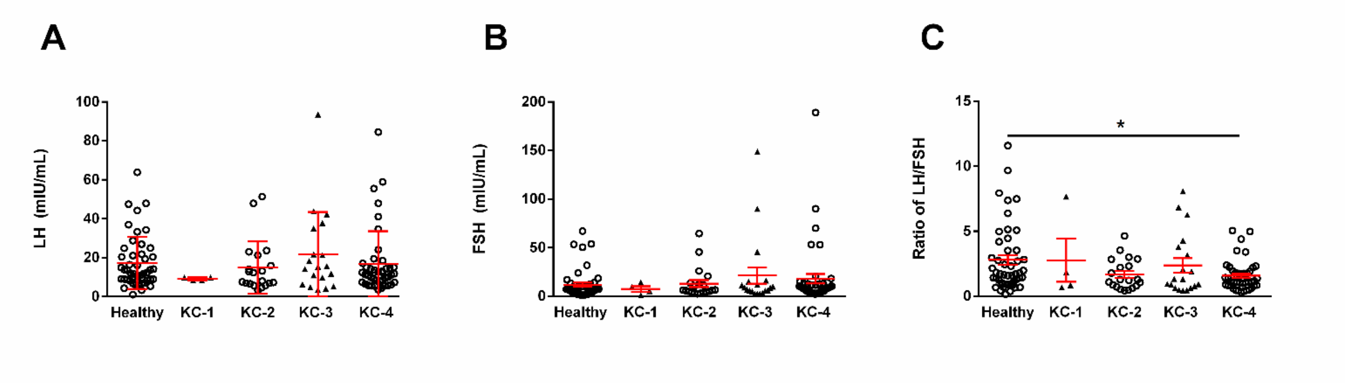

Figure S5. Severity-dependent expression of LH, FSH, and LH/FSH ratio in Healthy (n=47) and KC blood (plasma) samples. Severity grades were defined based on the Kmax: KC-1 (n=4), KC-2 (n=20), KC-3 (n=19) and KC-4 (n=43). (**A**) LH levels in Healthy and KCs, across all severities, (**B**) FSH levels in Healthy and KCs, across all severities, and (**C**) LH/FSH ratio in Healthy and KCs, across all severities. *p<0.05.

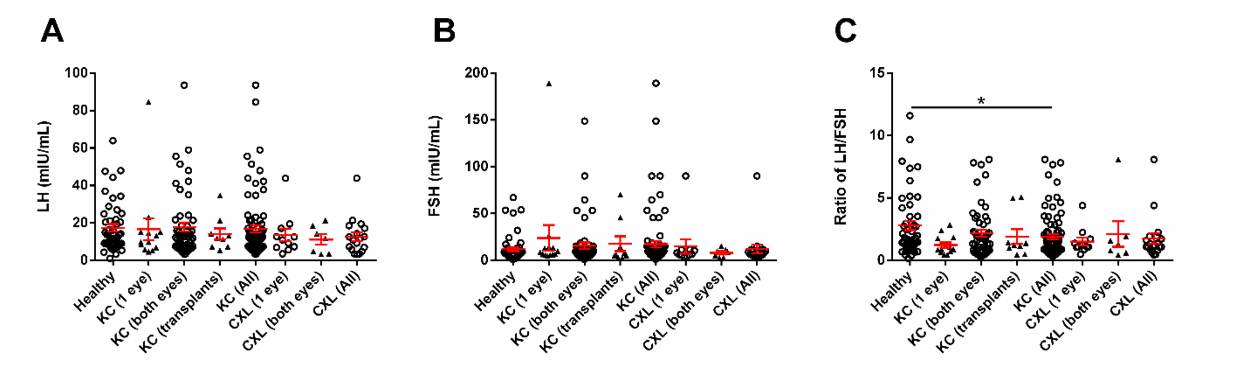

Figure S6. Expression of LH, FSH, and LH/FSH ratio in Healthy (n=47) and KC blood (plasma) samples. Seven different groups were compared: 1) KC on one eye (n=13), 2) KC on both eyes (n=53), 3) KCs with corneal transplants (n=9), 4) All KCs independent of treatment(s) (n=86), 5) KCs with collagen crosslinking on one eye (n=11), and 6) KCs with collagen crosslinking on both eyes (n=7), and 7) All KCs with collagen crosslinking treatment, independent of the number of eyes (n=18). (**A**) LH levels, (**B**) FSH levels, and (**C**) LH/FSH ratio. *p<0.05.

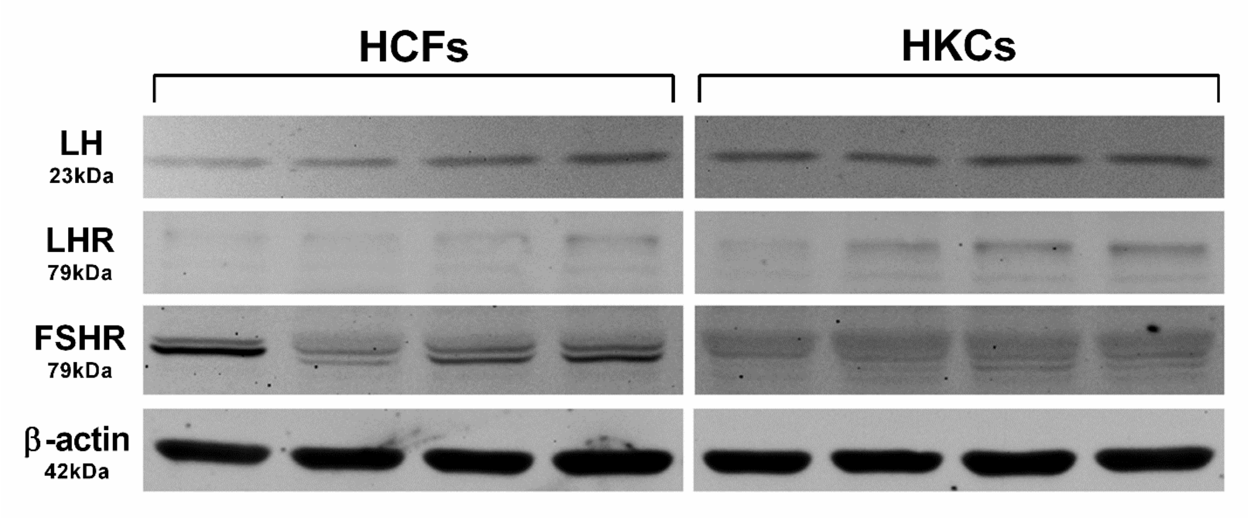

Figure S7. Representative Western Blots of HCF and HKC protein expression of LH, LHR, FSHR, and β-actin.

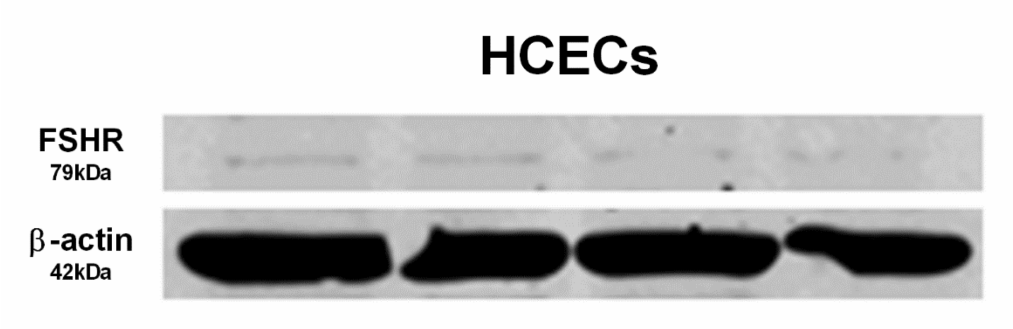

Figure S8. Representative Western Blots of HCEC protein expression of FSHR and β-actin.
